# Sustained endocrine profiles of a girl with WAGR syndrome

**DOI:** 10.1186/s12881-017-0477-5

**Published:** 2017-10-23

**Authors:** Yui Takada, Yasunari Sakai, Yuki Matsushita, Kazuhiro Ohkubo, Yuhki Koga, Satoshi Akamine, Michiko Torio, Yoshito Ishizaki, Masafumi Sanefuji, Hiroyuki Torisu, Chad A. Shaw, Masayo Kagami, Toshiro Hara, Shouichi Ohga

**Affiliations:** 1 Department of Pediatrics, Graduate School of Medical Sciences, Kyushu University, Fukuoka, 812-8582 Japan; 2 Section of Pediatrics, Department of Medicine, Division of Oral & Medical Management, Fukuoka Dental College, Fukuoka, 814-0193 Japan; 3 Department of Molecular and Human Genetics, Baylor College of Medicine, Houston, 77030 USA; 4 Department of Molecular Endocrinology, National Research Institute for Child Health and Development, Tokyo, 157-8535 Japan; 5 Present address: Fukuoka Children’s Hospital, Fukuoka, 813-0017 Japan; 6 Present address: Department of Pediatrics, National Hospital Organization Kyushu Medical Center, Fukuoka, Japan

**Keywords:** Wilms tumor, Aniridia, Genitourinary anomalies and mental retardation (WAGR) syndrome, Epigenetics, Neuroendocrine function, Methylation

## Abstract

**Background:**

Wilms tumor, aniridia, genitourinary anomalies and mental retardation (WAGR) syndrome is a rare genetic disorder caused by heterozygous deletions of *WT1* and *PAX6* at chromosome 11p13*.* Deletion of *BDNF* is known eto be associated with hyperphagia and obesity in both humans and animal models; however, neuroendocrine and epigenetic profiles of individuals with WAGR syndrome remain to be determined.

**Case presentation:**

We report a 5-year-old girl with the typical phenotype of WAGR syndrome. She showed profound delays in physical growth, motor and cognitive development without signs of obesity. Array comparative genome hybridization (CGH) revealed that she carried a 14.4 Mb deletion at 11p14.3p12, encompassing the *WT1*, *PAX6* and *BDNF* genes. She experienced recurrent hypoglycemic episodes at 5 years of age. Insulin tolerance and hormonal loading tests showed normal hypothalamic responses to the hypoglycemic condition and other stimulations. Methylation analysis for freshly prepared DNA from peripheral lymphocytes using the pyro-sequencing-based system showed normal patterns of methylation at known imprinting control regions.

**Conclusions:**

Children with WAGR syndrome may manifest profound delay in postnatal growth through unknown mechanisms. Epigenetic factors and growth-associated genes in WAGR syndrome remain to be characterized.

**Electronic supplementary material:**

The online version of this article (10.1186/s12881-017-0477-5) contains supplementary material, which is available to authorized users.

## Background

Wilms tumor, aniridia, genitourinary anomalies and mental retardation (WAGR) syndrome is a rare genetic disorder caused by the chromosomal defect at 11p13.3 [1]. Genetically, segmental deletions encompassing the *WT1* and *PAX6* genes are known to cause the syndromic phenotype, while an accompanying phenotype of obesity is linked to the extensive deletion involving the *brain-derived neurotrophic factor* (*BDNF*) locus [2]. Thus, patients with the WAGR and obesity (O) phenotypes are designated as having WAGRO syndrome [2].

Imprinting-associated disorders commonly present with abnormal physical growth and autism. Prader-Willi syndrome is an example of such diseases that are phenotypically characterized by muscular hypotonia in infancy, short stature and hyperphagic behavior with cognitive impairment later in childhood [3]. Although autistic and hyperphagic behaviors are known to overlap between WAGRO and Prader-Willi syndromes, no information is currently available for epigenetic impacts of BDNF insufficiency on gene expression in the developing brain of humans and animals. This study was thus directed to test whether WAGRO syndrome might have deregulated conditions in neuroendocrine or methylation profiles.

## Case presentation

A 1-year and 10-month-old girl was referred to our department for assessment of growth delay and absence of speech. She was born at the 41st week of gestational age with birth weight of 2950 g. Congenital aniridia, cataracts and macular hypoplasia were noticed at birth. Genitourinary apparatus was normal in appearance.

When she was first referred to our department at 1 year and 10 months of age, her height (74.4 cm, −2.8SD) and weight (8.5 kg, −3.3SD) were evaluated to be small for age. At this age, her motor skills and cognitive development were considered as low as 10 months of age. She developed bilateral renal tumors at 2 years and 1 month of age, for which surgical resection was conducted. Pathological examination confirmed the diagnosis of Wilms tumor. The renal tumors responded favorably to standard regimens of chemotherapy. She was well nourished without recurrence of tumors for over 4 years after the chemotherapy. Thus, the clinical features of this case fit to the typical phenotypes of WAGR syndrome, as previously reported [1, 2, 4].

**Fig. 1:**
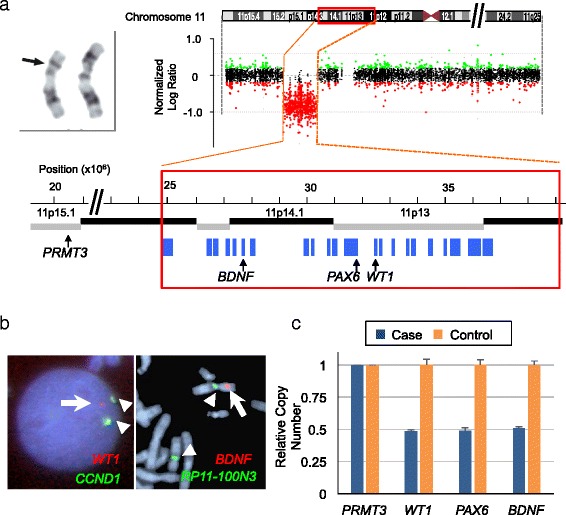
Genetic analyses of the present cases. **a** Array-CGH data for the present case. The left-top panel depicts the abnormal G-band pattern (arrow) of chromosome 11 in the peripheral leukocytes. On the right top, the log_2_ values of relative copy numbers are plotted against the base position (x10E6) at chromosome 11. Color codes represent gain (green) or loss (red) of the probed regions. The horizontal dashed lines indicate the thresholds of duplication (0.5) and heterozygous deletion (−1.0). The lower-panel shows a magnified view of the deleted region. G-band locus (thick, black and gray lines) and protein-coding genes (blue boxes) are mapped on the coordinates available at the UCSC genome browser (GRCh37/hg19). Chromosomal locations of the three WAGR syndrome-associated genes (*BDNF*, *PAX6* and *WT1*) are denoted under the diagram. **b** The FISH image. The green (arrowheads) and red (arrows) signals show *CCND1* (control) and *WT1* loci, respectively. Note that one copy of the *WT1* (arrow) is missing while the two copies of *CCND1* signals (arrowheads) are unaffected. **c** Quantitative PCR data for this case and a healthy control. *PRMT3* (chr11:20,409,076–20,530,879) serves as an internal control. Bar plots show the relative copy numbers of *WT1, PAX6* and *BDNF* (mean ± SD, *n* = 4) to that of *PRMT3* for each individual

The G-band test revealed the karyotype of 46,XX,del(11)(p11.2p14) (Fig 1a). An array-comparative genome hybridization analysis (Clinical Microarray ver. 8.1 at Baylor MGL), and found the 14.5-Mb deletion at 11p14.3p12 (Fig 1a). The minimal segment of deletion were defined as arr[GRCh37] 11p14.3p12(24,792,569_39,222,929)×1. According to the GRCh37/hg19 assembly, the deleted region was located at the chromosomal band, 11p14.3p12. The deleted interval involved 50 genes in the adjacent loci, including *PAX6* (chr11:31,806,340–31,839,509) and *WT1* (chr11:32,409,322–32,457,081). The heterozygous deletions of *WT1* and *BDNF* were validated with FISH and qPCR analyses (Fig. 1b, c; see Additional file 1). We further excluded that this patient had a variant coding sequence of *BDNF* at the non-deleted allele (data not shown). Together, these data indicate that the heterozygous deletion of *BDNF*, but not a hypomorphic mutation, such as Val66Met variant [5], caused the developmental delay in this patient. Because we were not allowed to analyze the parental chromosomes, it remained to be determined whether the deletion occurred de novo or as the consequence of chromosomal defects with lower penetrance, such as balanced translocations.

At 5 years of age, she reached 91.3 cm (−3.5 SD) of height and 12.45 kg (−2.2 SD) of body weight. She had recurrent episodes of ketogenic hypoglycemia on starvation. To assess whether she may have neuro-endocrine dysfunction, we conducted a series of hormonal loading tests. Insulin tolerance test (0.1 U/kg) revealed that her blood glucose rapidly declined to 35 mg/dl after infusion, while growth hormone (GH), adrenocorticotropic hormone (ACTH) and cortisol were all normally increased in response to the hypoglycemic condition at 15–30 min (Additional file 1: Figure S2A). Combined infusion of 500 μg/kg L-arginine, 1.5 μg/kg CRH and 10 μg/kg TRH induced normal patterns of release in corresponding hormones (Additional file 1: Figure S2B). Oral glucose tolerance test with 1.75 g/kg of glucose resulted in normally fluctuated blood glucose and insulin levels, while lactate and pyruvate did not show abnormal patterns of accumulation (Additional file 1: Figure S2C). These data reassured that she had no obvious predisposing causes for hypoglycemia. She was likely intolerant of starved conditions owing to immature energy homeostasis as frequently observed in preschool children, but not as a consequence of WAGR-relevant phenotype.

This case had typical growth and neurodevelopmental profiles as WAGR syndrome with large chromosomal deletions (Table 1) [1]. We therefore considered it valuable to characterize her epigenetic profiles, which might disclose one of molecular mechanisms for delays in postnatal growth and development in WAGR syndrome. Recent studies indicated that chromosomal defects in the WAGR-critical region encompassed the *LGR4* gene [6]. The heterozygous deletion of *LGR4* was reported to cause the secondary effects on the epigenetic status through disturbing the expression of chromatin modifier proteins. Towards this end, we investigated whether children with WAGR syndrome might bear aberrant methylation profiles on previously known imprinting regions. However, this case had normal methylation patterns at known loci for imprinting-associated disorders when using lymphocytes (Additional file 1: Table S1) [7]. CARE guidelines were followed in reporting this case.

**Table 1:** Phenotypic spectrum of WAGR syndrome with 10 Mb or larger deletions

No	Case ID	Age	Sex	Reference (Author, Year)	Cytoband	Size (Mb)	Genome Assembly	Start point	End point	Height	Body weight	Neurological Signs^*a*^
1	F24a	16	F	Xu S, 2008 [4]	11p12p15.1	23.0	GRCh 35 (hg17)	20,040,419	43,071,919	NA	NA	ID, OCD
2	F8a	11	F	Xu S, 2008 [4]	11p14p12.3	19.1	GRCh 35 (hg17)	25,086,501	44,212,278	NA	NA	ID, OCD, ASD
3	NIH10	6	M	Xu S, 2008 [4]	11p11.2p14.1	18.2	GRCh 35 (hg17)	27,656,700	45,858,752	NA	NA	DD
4	F17a	36	F	Xu S, 2008 [4]	11p14p12.3	15.6	GRCh 35 (hg17)	25,086,501	40,651,423	NA	NA	ID, ASD, ADHD, OCD, Anxiety
5	NIH3	4	M	Xu S, 2008 [4]	11p14p12.3	15.3	GRCh 35 (hg17)	24,202,909	39,529,029	NA	NA	DD
6	F10a	7	F	Xu S, 2008 [4]	11p11.2p13	14.9	GRCh 35 (hg17)	31,284,414	46,194,871	NA	NA	ID, DD, OCD, Anxiety
7	F20a	15	M	Xu S, 2008 [4]	11p14p12.3	14.6	GRCh 35 (hg17)	25,844,571	40,482,022	NA	NA	ID, DD, ASD, ADHD, OCD, Anxiety
8	YS 013	5	F	[This study]	11p14.3p12	14.4	GRCh37 (hg19)	24,792,569	39,222,929	91.3 cm (−3.5 SD)	12.45 kg (−2.2 SD)	DD, ASD, Hyperphagia
9	NIH9	7	F	Xu S, 2008 [4]	11p13p15.1	14.4	GRCh 35 (hg17	20,759,560	35,124,532	NA	NA	ID, ASD
10	F7a	13	F	Xu S, 2008 [4]	11p14p12.3	14.3	GRCh 35 (hg17	24,542,321	38,824,714	NA	NA	ASD, DD, ADHD
11	NIH6	17	M	Xu S, 2008 [4]	11p14p12.2	14.2	GRCh 35 (hg17	26,005,134	40,174,102	NA	NA	ID, ASD, ADHD, Anxiety
12	NIH2	19	M	Xu S, 2008 [4]	11p13p15.1	13.5	GRCh 35 (hg17	20,135,621	33,614,001	NA	NA	ID, ASD
13	F3a	26	F	Xu S, 2008 [4]	11p12p13	12.9	GRCh 35 (hg17	31,284,414	44,212,278	NA	NA	ID, DD, Seizure, Depression
14	F21a	12	F	Xu S, 2008 [4]	11p14p12.3	12.5	GRCh 35 (hg17)	25,336,593	37,873,278	NA	NA	ADHD, OCD, Anxiety
15	F14a	12	F	Xu S, 2008 [4]	11p14p12.1	12.2	GRCh 35 (hg17)	28,001,853	40,239,548	NA	NA	ID, ASD, OCD, Anxiety, ADHD
16	F1a	11	M	Xu S, 2008 [4]	11p13p14.3	11.4	GRCh 35 (hg17)	24,848,855	36,266,146	NA	NA	ID, ASD, Anxiety, OCD
17	NIH11	4	M	Xu S, 2008 [4]	11p14p12.2	10.7	GRCh 35 (hg17)	26,690,778	37,341,623	NA	NA	ID, ASD
18	F23a	16	M	Xu S, 2008 [4]	11p14p12.1	10.6	GRCh 35 (hg17)	27,675,634	38,235,380	NA	NA	ID, ASD, DD, OCD
19	Patient 2	2	F	Yamamoto T, 2013 [13]	11p13p12	10.5	GRCh 35 (hg17)	32,990,627	43,492,580	75.6 cm (−3.5SD)	11.9 kg (+0.8SD)	DD
20	NIH1	26	M	Xu S, 2008 [4]	11p14p12.1	10.2	GRCh 35 (hg17)	27,692,635	37,916,281	NA	NA	ID, ASD
21	F19a	5	M	Xu S, 2008 [4]	11p14p12.2	10.1	GRCh 35 (hg17)	26,699,475	36,757,882	NA	NA	ID, DD, ASD

^a^ *Abbreviations*: *ID* intellectual disability, *OCD* obsessive compulsive disorder, *ASD*, autism spectrum disorder, *DD*, developmental delay, *ADHD*, attention deficit hyperactive disorder

## Discussion and conclusions

We described a girl with typical phenotypes of WAGR syndrome. By defining the deleted interval in the short arm of chromosome 11, we confirmed that she had heterozygous deletions of *WT1*, *PAX6* and *BDNF1*. The *BDNF* gene encodes brain-derived neurotrophic factor, one of the most potent activators of neurogenesis and synaptogenesis in the central nervous system [8]. Precise mechanisms for developing the obesity phenotype with *BDNF* deletion remain to be elusive; however, hypothalamic dysfunctions due to hypomorphic mutation of *BDNF* have been proposed in both humans and animal models [9, 10]. Although our case did not show hyperphagic behaviors during this study period, her metabolic status may be deranged to drive this phenotype in later stages.

BDNF and its cognate receptor, tyrosine receptor kinase B (TrkB), coordinate glucose metabolism and energy homeostasis in the hypothalamus [11, 12]. Because we were unable to detect hyper- or hypo-functioning responses to hormonal loading tests in this case, she was likely to experience hypoglycemic episodes on starvation without specific causes in the neuro-endocrine system. However, one could still argue that such hypothalamic dysfunctions might be evident only when she develops obesity. Thus, serial observation of physical growth and its correlation with hyperphagic and autistic behaviors in childhood through adolescence will be necessary to clarify the neural bases for obese phenotype of WAGR syndrome.

We further verified that she had unaltered methylation profiles at the CpG sites of previously known imprinting genes. Although it still remains unknown why the segmental deletion at 11p15 lead to short stature in WAGR syndrome in early childhood, these data provide a clue for directing future epigenetic study on WAGR syndrome. Consecutive evidence in future studies will warrant these data and provide insight into genotype-phenotype correlations for children with WAGR syndrome.

## Additional file

Additional file 1: For cytogenetics, quantitative PCR for copy number and gene expression analyses, methylation analysis, and ethics statement. Figure S1. Neuro-endocrine profile of the present case. Table S1. Methylation profile of the present case. Additional file References 1–3. (PDF 652 kb)

## Abbreviations

a-CGH: Array-based Comparative Genome Hybridization
BDNF: Brain-derived neurotrophic factor
CNV: Copy Number Variation
qPCR: Quantitative Polymerase Chain Reaction
WAGR: Wilms tumor, aniridia, genitourinary anomalies and mental retardation
