# Supplementary material for: Sustained endocrine profiles of a girl with WAGR syndrome
Source: BMC Med Genet. 2017 Oct 23;18:117. doi: 10.1186/s12881-017-0477-5 (PMC5654094; doi:10.1186/s12881-017-0477-5)
Supplement: Additional file 1: — For cytogenetics, quantitative PCR for copy number and gene expression analyses, methylation analysis, and ethics statement. Figure S1. Neuro-endocrine profile of the present case. Table S1. Methylation profile of the present case. Additional file References 1–3. (PDF 652 kb) [file 12881_2017_477_MOESM1_ESM.pdf]

Supplementary Materials for

Sustained endocrine and methylation profiles of a girl with WAGR syndrome

Yui Takada, Yasunari Sakai, Yuki Matsushita, Kazuhiro Ohkubo, Yuhki Koga, Satoshi Akamine, Michiko Torio, Yoshito Ishizaki, Masafumi Sanefuji, Hiroyuki Torisu, Chad A Shaw, Masayo Kagami, Toshiro Hara

This file contains the following items:

- Supplementary methods
- Table S1
- Fig S1
- Supplementary References

Supplementary Methods

Cytogenetics

Conventional technique of fluorescence *in situ* hybridization (FISH) was applied as previously described [1]. Briefly, RP1-74J1 (chr11:32,383,408-32,510,987) and 32-132011 (Vysis/Abbott Laboratories) were selected to probe *WT1* and *CCND1*, respectively. Spectrum Orange and Green (Chroma Technology Corp) were used to label these probes, and the probes were co-hybridized with fixed lymphocytes.

Copy number and gene expression analyses

Standard protocols for microarray-based comparative genome hybridization (a-CGH) were used as previously described [1, 2]. The coordinates of genes and chromosomal breakpoints were defined according to the GRCh37/hg19 assembly at the UCSC genome browser (http://genome.ucsc.edu/). Quantitative (q) PCR assays were carried out with the SYBR Green system (ABI) according to the manufacturer's protocol. Oligonucleotide primers were designed with the Primer3 software (http://bioinfo.ut.ee). The primer sequences (5' to 3') used in this study were: CCAGGCCCAACAAGAATCAC and GTGAGAAGCTGGACCTCTGT (*WT1*); CATGAGGTGAGGCAAGCATC and AGGCTCTTGGTTCTGAAGCT (*PAX6*); GCCATATAGCCTCTGTCCCA and GCCCATAGTCAAATCCAGCC (*PRMT3*); TTCCCCTTTTAATGGTCAATG and GAACTCCCAGTGCCGAACTA (*BDNF*).

Methylation analysis

Two μg of genomic DNA from the patient's leukocytes was treated with bisulfite. Differentially methylated regions with multiple CpG sites were PCR-amplified with the region-specific primer sets, as previously described [3]. The yielded PCR amplicons were then subjected to pyrosequencing analysis using PyroMark Q24 (Qiagen, Valencia, CA, USA). Methylation indexes were obtained accordingly, and the values for the CpG dinucleotide (CpG #7 in *PLAGL1*, **Supplementary Table 1**) was omit from analysis because it was located on a polymorphic site.

Ethics statement

This study was approved by the institutional review board at Kyushu University (#461-00) and conducted with stringent compliance to the guidelines for genetic and clinical studies. Written informed consent was obtained from the parents for publication of this report.

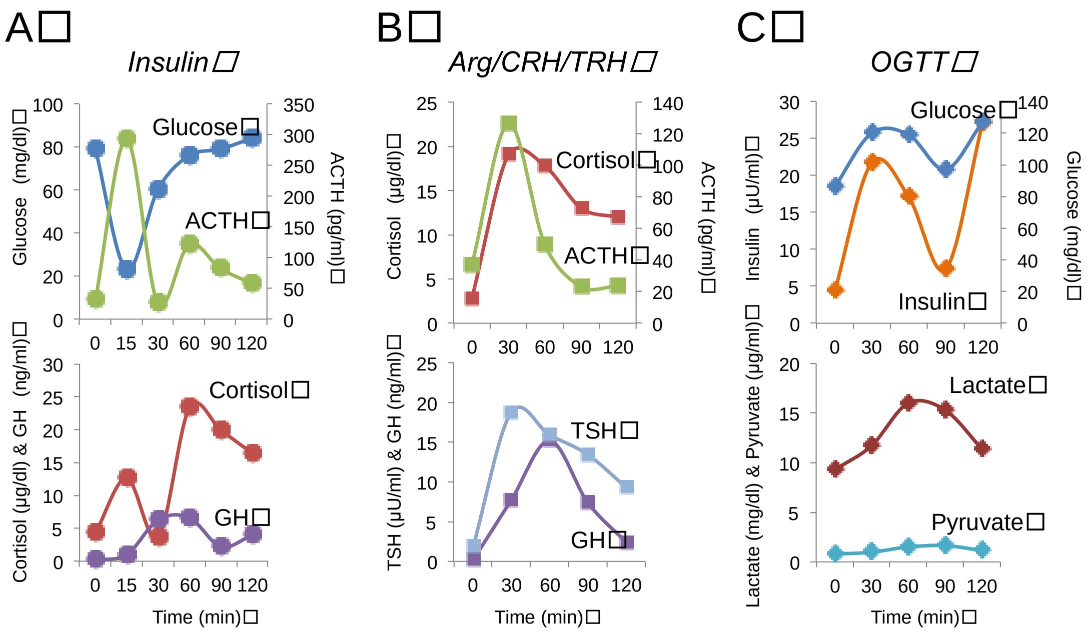

Fig S1. Neuro-endocrine profile of the present case

Time courses of indicated peptides are plotted as results of insulin tolerance (A), combined infusions of L-arginine, CRH and TRH (B), and oral glucose tolerance tests (C). Note that the present case cleared the diagnostic cut-off levels of ACTH and cortisol (3-fold increase), GH (≥ 6.0 ng/ml) and TSH (robust increase after stimulations with no more than 25 µU/ml of the peak value). No abnormal accumulation of the glucose, lactate or pyruvate was noted.

Supplementary Table 1. Methylation profile of the present case

Gene Symbol (Alias)	Band Locus	Phenotype & Disease (MIM ID)	CpG site	Methylation index (%)				
				This case	Control			
					n	Median	min	Max
H19	11p15.5	SRS[a]	1	63	59	57	51	67
		(103280)	2	62	59	56	45	64
			3	58	59	52	43	61
			4	58	59	51	43	58
			5	53	59	49	38	57
			6	50	59	47	37	55
MEST	7q32.2	SRS	1	58	55	60	56	70
(PEG1)		(601029)	2	57	55	59	55	69
			3	53	55	58	52	68
			4	59	55	61	42	73
			5	58	55	57	47	66
			6	61	55	60	54	70
PEG10	7q21.3	SRS	1	52	48	56	50	59
		(609810)	2	55	48	55	50	59
			3	51	48	53	48	59
			4	55	48	54.5	50	59
			5	49	48	50	45	55
KCNQ1	11p15.5-p15.4	BWS[b]	1	56	53	58	49	66
		(607542)	2	56	53	61	52	68
			3	50	53	48	41	54
			4	51	53	48	42	55
			5	58	53	67	55	72
			6	59	53	64	55	71
PLAGL1/ZAC1	6q24.2	TNDM[c]	1	45	49	47	33	52
		*603044	2	45	49	46	34	51
			3	52	49	48	40	56

(Supplementary Table 1, continued)

PLAGL1/ZAC1			4	40	49	39	31	47
			5	49	49	50	44	58
			6	52	49	49	41	55
			7			*NA*[h]		
			8	56	49	53	47	58
SNRPN	15q11.2	PWS/AS[d]	1	53	60	55	50	60
		(182279)	2	50	60	49	44	54
			3	52	60	51	46	57
			4	51	60	52	47	57
			5	60	60	63	58	68
GNAS	20q13.32	PHP[e]	1	43	45	41	34	45
		(139320)	2	44	45	40	33	45
			3	45	45	43	35	47
			4	43	45	38	32	42
			5	45	45	40	33	45
			6	41	45	38	31	42
			7	46	45	40	32	44
MEG3	14q32.2	KOS[f]	1	44	55	52	43	56
		(608149)	2	46	55	55	52	65
		TS[g]	3	45	55	37	32	55
		(616222)	4	51	55	60	44	74
			5	40	55	36	26	47
IG	14q32.2	KOS	1	59	56	58	49	68
		(608149)	2	56	56	54	40	62
		TS	3	66	56	68	54	78
		(616222)	4	54	56	53.5	43	64

[a]SRS, Silver-Russell syndrome; [b]BWS, Beckwith-Wiedeman syndrome; [c]TNDMc, transient neonatal diabetes mellitus; [d]PWS/AS, Prader-Willi syndrome/Angelman syndrome ;[e]PHP, pseudohypoparathyroidism; [f]KOS, Kagami-Ogata syndrome; [g]TS, Temple syndrome; and [h]NA, not available.

Supplementary References

1. Sakai Y, Ohkubo K, Matsushita Y, Akamine S, Ishizaki Y, Torisu H, Ihara K, Sanefuji M, Kim MS, Lee KU *et al*: **Neuroendocrine phenotypes in a boy with 5q14 deletion syndrome implicate the regulatory roles of myocyte-specific enhancer factor 2C in the postnatal hypothalamus**. *European journal of medical genetics* 2013, **56**(9):475-483.
2. Sakai Y, Shaw CA, Dawson BC, Dugas DV, Al-Mohtaseb Z, Hill DE, Zoghbi HY: **Protein interactome reveals converging molecular pathways among autism disorders**. *Science translational medicine* 2011, **3**(86):86ra49.
3. Kagami M, Kato F, Matsubara K, Sato T, Nishimura G, Ogata T: **Relative frequency of underlying genetic causes for the development of UPD(14)pat-like phenotype**. *European journal of human genetics : EJHG* 2012, **20**(9):928-932.
